# Validation of the Southampton Difficulty Scoring System in Robotic Liver Surgery and Development of the International RoboLiver Difficulty Scoring System

**DOI:** 10.1097/XCS.0000000000001978

**Published:** 2026-05-01

**Authors:** Soufyan El Adel, Gabriela Pilz da Cunha, Rong Liu, Iswanto Sucandy, Fabrizio Di Benedetto, Mathieu D’Hondt, Dionisios Vrochides, Roland Croner, Ernesto Sparrelid, Francesca Ratti, David Geller, Tom K. Gallagher, Brian KP Goh, Elio Jovine, Jawad Ahmad, Andrea Ruzzenente, Ricardo Robles Campos, Steven White, Mikhail Efanov, Ricardo Memeo, Umberto Cillo, Mohammed Alzoubi, Moritz Schmelzle, Adnan Alseidi, Susan van Dieren, Rutger-Jan Swijnenburg, Mohammad Abu Hilal

**Affiliations:** From the Department of Surgery, Amsterdam UMC, University of Amsterdam, the Netherlands (El Adel, Pilz da Cunha, van Dieren, Swijnenburg); Cancer Center Amsterdam, the Netherlands (El Adel, Pilz da Cunha, Swijnenburg); Department of Surgery, University of Jordan, Amman, Jordan (El Adel, Alzoubi, Abu Hilal); Faculty of Hepatopancreaotbiliary Surgery, the First Medical Center of Chinese PLA General Hospital, Beijing, China (Liu); Advent Health Tampa, Digestive Health Institute, Tampa, FL (Sucandy); Hepato-Pancreato-Biliary Surgery and Liver Transplantation Unit, University of Modena and Reggio Emilia, Modena, Italy (Di Benedetto); Department of Digestive and Hepatobiliary Pancreatic Surgery, Groeninge Hospital, Kortrijk, Belgium (D’Hondt); Division of Abdominal Transplantation, Department of Surgery, Carolinas Medical Center, Atrium Health, Charlotte, NC (Vrochides); Department of General, Visceral, Vascular, and Transplant Surgery, Otto-von-Guericke University Hospital, Magdeburg, Germany (Croner); Division of Surgery, Department for Clinical Science, Intervention and Technology (CLINTEC), Karolinska Institute, Karolinska University Hospital, Stockholm, Sweden (Sparrelid); Hepatobiliary Surgery Division, IRCCS, San Raffaele Hospital, Milan, Italy (Ratti); Department of Surgery, Division of HPB Surgery, University of Pittsburgh, Pittsburgh, PA (Geller); St. Vincent’s University Hospital, Elm Park, Dublin, Ireland (Gallagher); Department of Hepatopancreatobiliary and Transplant Surgery, Singapore General Hospital, National Cancer Center Singapore, and Duke-National University of Singapore Medical School, Singapore, Singapore (Goh); Alma Mater Studiorum, University of Bologna, AOU Sant’Orsola Malpighi, IRCCS at Maggiore Hospital, Bologna, Italy (Jovine); University Hospital Coventry and Warwickshire, Coventry, United Kingdom (Ahmad); Department of Surgery, University of Verona, Verona, Italy (Ruzzenente); Department of General, Visceral, and Transplantation Surgery, Clinic and University Hospital Virgen de la Arrixaca, IMIB-ARRIXACA, El Palmar, Murcia, Spain (Campos); Department of Surgery, Newcastle upon Tyne Hospitals NHS Foundation Trust, Newcastle upon Tyne, United Kingdom (White); Department of Hepato-Pancreato-Biliary Surgery, Moscow Clinical Scientific Center, Moscow, Russia (Efanov); Hepato-Pancreato-Biliary Surgery Unit, Miulli Hospital, Acquaviva delle Fonti, Bari, Italy (Memeo); Department of Surgery, Oncology and Gastroenterology, Hepatobiliary Surgery and Transplantation Unit, Padua University Hospital, Padua, Italy (Cillo); Department of General, Visceral and Transplant Surgery, Hannover Medical School, Hannover, Germany (Schmelzle); Departments of Surgery (Alseidi), University of California, San Francisco, CA; Medical Education (Alseidi), University of California, San Francisco, CA; Department of Surgery, Southampton General Hospital, Southampton, United Kingdom (Abu Hilal).

## Abstract

**BACKGROUND:**

Robotic liver surgery (RLS) provides technical advantages over laparoscopic liver surgery, but no validated robotic-specific difficulty scoring system (DSS) exists. We evaluated the applicability of the Southampton DSS to RLS and developed a dedicated RLS difficulty model.

**STUDY DESIGN:**

This multicenter retrospective cohort study included adults undergoing planned RLS across 24 international hepatobiliary centers. The Southampton DSS was assessed for calibration and discrimination in predicting intraoperative complications. Given limited performance, a robotic-specific model (International RoboLiver DSS) was developed using multivariable logistic regression with prolonged operative time (more than 280 minutes; 75th percentile) as a surrogate of technical difficulty. Model discrimination, calibration, and bootstrap internal validation were performed.

**RESULTS:**

Among 1,497 RLS patients, higher Southampton DSS categories were associated with increased intraoperative complications (p = 0.003); however, discrimination was poor (area under the curve [AUC] 0.571, 95% CI 0.530 to 0.612) with miscalibration (slope 0.43; intercept 0.06). Independent predictors of prolonged operative time included neoadjuvant chemotherapy, earlier extrahepatic surgery, lesion greater than 50 mm, multiple lesions, bilobar disease, and technically or anatomically major resection. The International RoboLiver DSS demonstrated moderate discrimination (AUC 0.719, 95% CI 0.686 to 0.751) with excellent calibration (intercept 0.00; slope 1.14). Bootstrap validation confirmed model stability (corrected AUC 0.719).

**CONCLUSIONS:**

Difficulty factors in RLS partially overlap with laparoscopic liver surgery but are not directly transferable. The International RoboLiver DSS provides a calibrated, robot-specific tool for preoperative complexity stratification and operative planning in RLS. External validation is required.

## Abstract 2

This multicenter study evaluating 1,497 robotic liver surgery (RLS) procedures found that the Southampton Laparoscopic Difficulty Score poorly predicts intraoperative complications in RLS. The International RoboLiver Score was developed using prolonged operative time as a surrogate for surgical difficulty, providing a more accurate measure of procedure complexity in RLS.

Minimally invasive liver surgery (MILS) has gained acceptance owing to its association with reduced blood loss, shorter hospital stay, and lower morbidity rates.^1^ Although laparoscopic liver surgery (LLS) is the primary minimally invasive approach, recent advances have led to the development of robotic liver surgery (RLS). RLS provides technical advantages including enhanced dexterity, 3-dimensional visualization, and ergonomic benefits. Studies comparing RLS and LLS have shown modest improvements in perioperative outcomes; however, the high cost of RLS remains an important consideration.^2-5^

The role of the RLS in complex liver resections, particularly in challenging anatomical areas, such as the posterosuperior segments, is not yet fully understood. Laparoscopic resections in the posterior segments have been considered complex resections to be performed only after a surgeon has mastered resections in the anterior segments and formal anatomical resections. This difficulty has been confirmed by the Southampton,^6^ IWATE,^7^ and Institut Mutualiste Mountsouris^8^ difficulty scoring systems (DSSs) for LLS. Retrospective studies have suggested that the benefits of RLS become more apparent with higher procedural complexity.^9,10^ However, stepwise progression is the key to ensuring patient safety during the initial phases of adopting a new technique, including the most advanced robotic systems. Hence, a robust DSS that can assist surgeons in selecting cases that align with their skill levels is needed, especially during their learning curve in performing robotic liver resections. This need is strongly recommended in the recent Brescia Internationally Validated European Guidelines on Minimally Invasive Liver Surgery,^11^ which state that “Difficulty scores are crucial for proper case selection,” however robot-specific DSS are lacking.

Although various difficulty classifications have been developed for LLS,^12^ relatively few studies have investigated the application of these scoring systems to RLS.^13-17^ To date, only 1 DSS has been specifically developed for RLS.^18^ However, this study used data from a single institution.

Given the inherent differences between RLS and LLS techniques, it is likely that each approach presents unique challenges that contribute to its level of difficulty. A single-center study by Sucandy and colleagues^19^ found that the Southampton laparoscopic liver difficulty score developed by Halls and colleagues^6^ is a strong predictor of intraoperative outcomes in RLS, yet has poor predictive ability for postoperative outcomes.

DSSs support both standardized reporting and safe stepwise adoption by enabling preoperative stratification of technical complexity. The Southampton DSS was developed for LLS using intraoperative complications as a marker of operative difficulty. However, the robotic platform may alter the frequency and nature of intraoperative events (including conversions), potentially reducing the sensitivity of this endpoint for difficulty discrimination in RLS. Therefore, in addition to evaluating the transferability of the Southampton DSS to RLS, we aimed to develop an RLS-specific DSS.

This study aimed to assess the applicability of the Southampton DSS for RLS in a large multicenter cohort of patients and to propose a novel DSS for RLS.

## METHODS

### Study design

This multicenter retrospective cohort study used data from 24 international hepatobiliary centers to validate the Southampton DSS. The centers provided prospectively collected standardized data for this study. Consecutive patients who underwent a planned robotic liver resection for benign and malignant liver lesions from the first implementation of RLS until December 2022 were included. Approval for data review and research was granted by the medical ethics committee of Amsterdam UMC (2025.0181).

Baseline characteristics and perioperative outcomes were compared across difficulty categories. The primary outcome measure was the incidence of intraoperative complications. Secondary outcomes were operative time, intraoperative blood loss, Pringle application, Pringle duration (when applied), conversion, hospital stay duration, overall morbidity, severe morbidity, and mortality. Calibration and discrimination of the Southampton DSS were assessed using intraoperative complications as primary markers for validation.

All adult patients (18 years or older) who underwent planned robotic liver resection for benign or malignant lesions were included. This also included procedures in which a combined robotic laparoscopic surgical approach was applied. Patients who underwent planned hand-assisted resections, major concurrent procedures (eg vascular reconstructions and colorectal, diaphragmatic, or pancreatic resections), nonformal liver resection (eg cyst fenestrations and biopsies), or resections for living donor hepatectomy were excluded.

### Surgical technique

A local multidisciplinary tumor board discussed all patients to assess the feasibility of local treatment with RLS, depending on disease characteristics, local expertise, and available resources. Most surgeries at each center were performed by a single surgeon, who led the robotic liver program. Hence, these cohorts represent procedures at all stages of the learning curve for RLS. The indications for RLS were similar in all institutions. Patient assignment to robotic surgery was determined at the discretion of the operating surgeon. Surgical technique and intraoperative management of RLS were performed in all centers according to the European expert consensus technique for robotic hepatectomy.^20^ Postoperative management protocols were performed according to the principles of Enhanced Recovery After Surgery at all centers.^21^

### The Southampton difficulty scoring system for laparoscopic liver surgery

The Southampton DSS categorizes patients into 4 distinct difficulty levels based on a point system correlated with the likelihood of intraoperative complications: low (less than or equal to 2), moderate,^3-5^ high,^6-9^ and extremely high.^10-15^ Points were assigned on the basis of the presence of specific patient and procedural characteristics (Table 1). This point system was used to classify patients undergoing RLS into 4 corresponding difficulty levels.

**Table 1.** Southampton Difficulty Scoring System Points System proposed by Halls et al^6^

Patient and procedure characteristic	Points assigned
Neoadjuvant chemotherapy	
No	0
Yes	1
Previous open liver resection	
No	0
Yes	5
Lesion type	
Benign	0
Malignant	2
Lesion size, cm	
<3	0
3–5	2
>5	3
Classification of resection	
Minor	0
Technically major	2
Anatomically major	4

### Definitions and outcomes

The data collected included patient demographics, medical and surgical history, tumor characteristics, operative details, postoperative outcomes, and in-hospital/90-day mortality. The liver segments were classified according to the Couinaud classification system.^22^ The Brisbane 2000 terminology was used to define the extent and type of liver resection performed, with a resection of at least 3 contiguous segments as major and those of less than 3 as minor.^23^ Minor liver resections in the anterolateral (segments 2, 3, 4b, 5, and 6) or posterosuperior (segments 1, 4a, 7, and 8) segments were defined as minor and technically major, respectively.^24,25^ Minor concurrent procedures included stoma creation or closure, hernia repair, lymphadenectomy, nephrectomy, adrenalectomy, oophorectomy, and thermal ablation. Intraoperative complications were defined according to the modified Satava classification, including incidents such as excessive bleeding, minor injury to adjacent organs (eg diaphragm, bowel, gallbladder), and conversion to open surgery. For excessive blood loss, 775 mL was used as the cutoff.^26^ Operative time was defined as the time in minutes between the first surgical incision and completion of wound closure. Prolonged operative time was defined as an operative time higher than the 75th percentile of the entire cohort (>280 minutes). Conversion was specifically defined as a change in the surgical approach from hand-assisted to hybrid or full laparotomy, with the exception of specimen extraction. Postoperative morbidity was based on the most severe complication and graded according to the Clavien-Dindo classification.^27^ Postoperative outcomes were reported during a follow-up period of 90 days.

### Statistical analysis

#### Validation of the Southampton difficulty scoring system

Categorical variables were presented as absolute numbers with percentages. Continuous nonnormally distributed variables were presented as medians with interquartile ranges. The baseline characteristics and perioperative outcomes of procedures of varying complexity, according to the Southampton DSS, were compared. Chi-square or Fisher’s exact tests were used, where appropriate, for the analysis of categorical data. The Kruskal-Wallis test was used to compare nonnormally distributed continuous data. The Jonckheere–Terpstra test was used to assess the presence of a monotonic trend across DSS categories for continuous outcomes and the Cochran-Armitage test for binary outcomes. A calibration curve was plotted to display the predicted vs observed risks of intraoperative complications per risk score. The calibration slope and intercept, along with their corresponding 95% CIs, were determined; a slope coefficient of 1 and an intercept of 0 represented perfect calibration. Goodness-of-fit was assessed using the Hosmer-Lemeshow test. The ability of the Southampton DSS point system to predict the primary outcome (intraoperative complications) was evaluated using receiver operating characteristic curve analysis, with the area under the curve (AUC) used to determine the model’s discrimination performance. An AUC value closer to 1 indicates a better model performance in distinguishing between the 2 outcomes. Brier’s score was used as an overall measure of accuracy. Patients with missing data for the baseline predictors used to calculate the difficulty score were excluded through list-wise deletion (n = 194), assuming that the pattern of missing data was random.

#### Development of a novel robotic liver surgery difficulty scoring system: the International RoboLiver difficulty scoring system

Due to the limited performance of the Southampton DSS in an RLS cohort, an RLS-specific DSS, termed the International RoboLiver DSS, was developed to enhance preoperative stratification of technical difficulty in RLS procedures. Factors potentially associated with operative difficulty and included as candidate predictors were selected a priori based on the literature and an international survey conducted among laparoscopic liver surgeons.^12,28^ To assess whether a robotic-specific DSS could be derived using the same endpoint as the Southampton DSS, we first performed binary logistic regression with intraoperative complications as the dependent variable, using the same dataset used for Southampton DSS validation. Predictors with a p value of <0.10 in univariable analysis were entered into a multivariable model, and associations were reported as adjusted odds ratios (aORs) with 95% CIs. Given that intraoperative complications yielded too few independently predictive factors to support a stable and clinically practical point-based DSS, we subsequently developed the International RoboLiver DSS using an otherwise identical modeling workflow (same candidate predictors and regression approach) but with prolonged operative time (greater than 75th percentile; more than 280 minutes) as the dependent variable. Prolonged operative time was selected as a pragmatic surrogate of technical difficulty because it reflects cumulative intraoperative complexity (eg exposure, vascular control, anatomic constraints, and extent of resection) and directly informs operating room planning.^29^ In contrast, postoperative outcomes such as morbidity and length of stay are strongly influenced by patient- and system-level factors that do not directly reflect intraoperative complexity and would introduce confounding; therefore, they were intentionally not used for score development. A sensitivity analysis was performed, excluding procedures performed during the learning curve (first 25 procedures per center). Points were assigned to each independently significant factor by dividing its coefficient by the coefficient of the binary categorical predictor with the smallest significant value (B_i_) and then rounding the result to the nearest whole number. The predicted risk based on this model was calculated using Equation 1:


$$Risk=\frac{1}{1+e^{-\left(intercept+B_{i}\times score\right)}}$$ (1)

where the intercept refers to the intercept of the logistic regression model, and scores the number of points. Thereafter, a point system was used to assign a score to each patient in the cohort. The proportion of patients with prolonged operative time, stratified by the number of points, was used to determine the observed risk. The number of points was plotted against the corresponding observed and predicted risk. The calibration of the points system was performed by applying a linear correction, deriving a calibration function by solving for *x* in the predicted risk equation, and substituting it into the observed risk equation. A calibrated function was used to calculate the predicted risk for each point’s total number. A linear function obtained graphically with a fixed intercept at 0 was used to determine the calibrated predicted risk for each number of points. The calibration curve was plotted and the slope and intercept along with their corresponding 95% CIs were determined. After calibration, the point system was divided into 4 categories based on the risk of prolonged operative time: low (less than 20%), medium (21% to 40%), and high (more than 40%). Its predictive ability was evaluated using a receiver operating characteristic curve analysis to determine the AUC. Internal validation of the difficulty score was performed using 1,000 bootstrap resamples. In each iteration, the model was refitted on a bootstrap sample and evaluated on the original dataset to estimate the bootstrap-corrected AUC and 95% bootstrapped CIs. Statistical significance was set at a p value of <0.050. All analyses were performed using R for Windows version 4.2.1 (R Foundation for Statistical Computing, Vienna, Austria).

## RESULTS

### Validation of the Southampton difficulty scoring system for robotic liver surgery

#### Baseline characteristics and outcomes across difficulty categories

Overall, 1,497 patients underwent RLS during the study period. Of these, 317 patients were classified as having low difficulty according to Southampton DSS, 613 as moderate, 546 as high, and 21 as extremely difficult. The baseline characteristics of each difficulty category are presented in **Supplemental Digital Content 1,** Table 1 (https://links.lww.com/JACS/A687). Across all Southampton DSS difficulty categories, the patients had a similar age and proportion of American Society of Anesthesiologists grade greater than or equal to 3. Higher difficulty scores had a higher proportion of male patients, had more often previously undergone previous extrahepatic abdominal surgery, had bigger largest lesion size, more disease with multiple lesions, and more technically major as well as anatomically major resections. Differences across the difficulty categories were also observed in terms of BMI, presence of cirrhosis, use of neoadjuvant chemotherapy, previous hepatic surgery, and presence of bilobar disease.

The short-term outcomes of each difficulty category are presented in Table 2. A significantly increasing monotonic trend was observed with higher procedural difficulty for intraoperative complications (p = 0.003). Additionally, a similar significant monotonic trend was observed for several secondary outcomes, including blood loss (p = 0.001), conversion rate (p = 0.006), Pringle use (p < 0.001), Pringle duration when applied (p = 0.001), intraoperative transfusion (p < 0.001), operative time (p = 0.001), hospital stay (p = 0.001), and severe morbidity (p<0.001). However, higher difficulty score was not associated with increased overall morbidity (p = 0.143), readmission (p = 0.092), in-hospital or 90-day mortality (p = 0.116), or R0 resection margin (p = 0.407).

**Table 2.** Short-Term Perioperative Outcomes Per Risk Category of the Southampton Difficulty Scoring System

Variable	Overall, N = 1,497	Low, n = 317	Moderate, n = 613	High, n = 546	Extremely high, n = 21	p Value for increasing monotonic trend
Intraoperative blood loss, mL, median (IQR)	100 (50–300)	50 (25–200)	100 (50–200)	150 (50–400)	400 (225–750)	<0.001^*^
Intraoperative complications, n (%)	224 (15.8)	37 (12.5)	83 (14.6)	97 (18.4)	7 (36.8)	0.003^*^
Conversion, n (%)	45 (3.0)	8 (2.5)	9 (1.5)	26 (4.8)	2 (9.5)	0.006^*^
Pringle application, n (%)	579 (38.8)	85 (26.9)	205 (33.6)	279 (51.1)	10 (47.6)	<0.001^*^
Pringle duration, min, median (IQR)	30 (20–45)	22 (16–30)	25 (18–39)	33 (21–49)	52 (45–62)	<0.001^*^
Intraoperative transfusion, n (%)	81 (5.5)	5 (1.6)	25 (4.1)	45 (8.3)	6 (30.0)	<0.001^*^
Operative time, min, median (IQR)	191 (138–280)	151 (115–207)	180 (129–244)	236 (175–346)	310 (260–450)	<0.001^*^
Hospital stay, d, median (IQR)	4 (3–6)	3 (2–5)	4 (3–5)	5 (3–7)	7 (5–12)	<0.001^*^
Overall morbidity, n (%)	286 (19.1)	59 (18.6)	106 (17.3)	114 (20.9)	7 (33.3)	0.143
Severe morbidity, n (%)	88 (5.9)	13 (4.1)	25 (4.1)	47 (8.6)	3 (14.3)	<0.001^*^
Readmission, n (%)	88 (6.0)	15 (4.7)	33 (5.5)	38 (7.2)	2 (9.5)	0.092
In-hospital/90-d mortality, n (%)	23 (1.6)	3 (1.0)	8 (1.3)	11 (2.0)	1 (4.8)	0.116
R0 resection margin for malignant cases only, n (%)	1,014 (90.2)	142 (92.2)	385 (88.7)	410 (90.1)	14 (77.8)	0.407

* Statistically significant.

IQR, interquartile range.

#### Calibration and discrimination of the Southampton difficulty scoring system for robotic liver surgery

**Figure 1.**
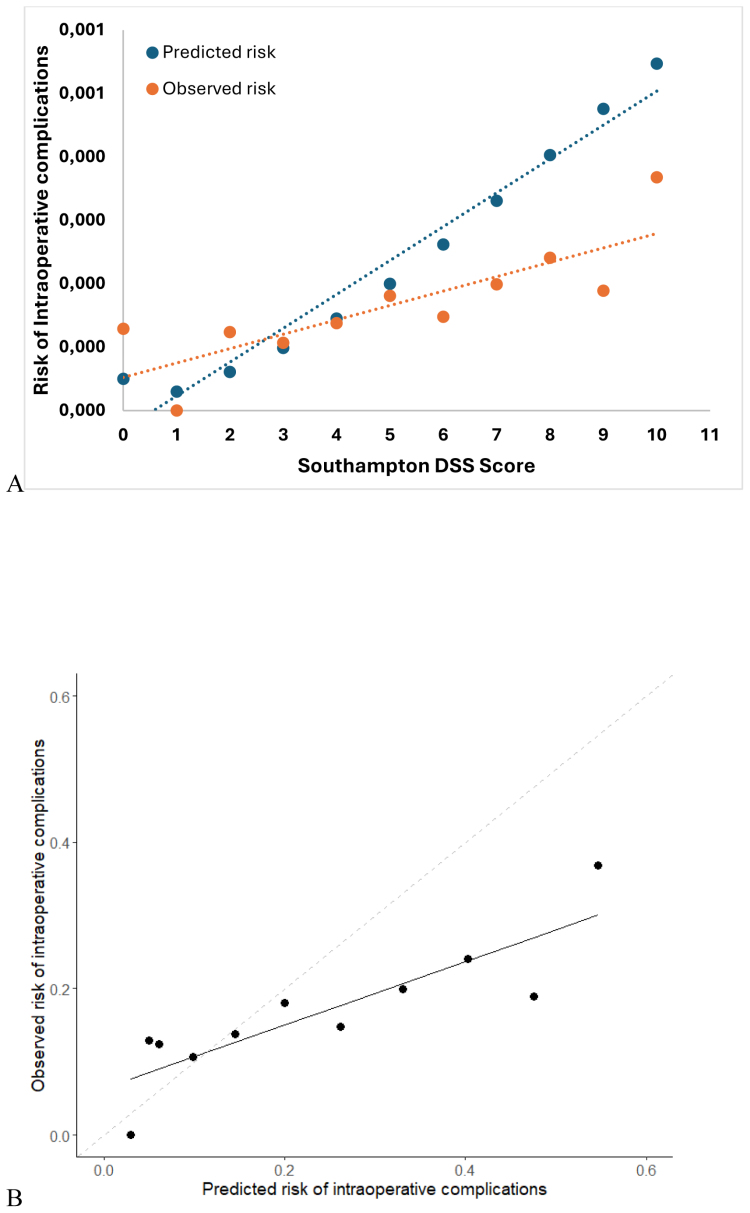
(A) Predicted and observed risk of intraoperative complications corresponding to each risk score of the Southampton difficulty scoring system (DSS). (B) Calibration plot of observed vs predicted risk based on the Southampton DSS.

The calibration plots are shown in Figure 1. Figure 1A shows an underestimation of the predicted risk in patients with lower difficulty scores (0, 2, and 3). For patients with a difficulty score of 1 or a difficulty score higher than 3, the Southampton DSS overestimated the actual risk of intraoperative complications. The calibration analysis demonstrated an intercept of 0.06 (95% CI 0.01 to 0.12), indicating that the model slightly underestimates risk on average. The calibration slope was 0.43 (95% CI 0.24 to 0.63), significantly less than 1, indicating that predicted probabilities were too extreme (overfitting) and suggesting miscalibration. The Hosmer-Lemeshow test was significant (p < 0.001), confirming a poor model fit. Brier’s score was 0.146, corresponding to a moderate predictive performance of the model. Discrimination analysis of Southampton DSS revealed an AUC of 0.571 (95% CI 0.530 to 0.612).

#### Development of the international RoboLiver difficulty scoring system

Only malignancy and lesions greater than 50 mm were identified as independent predictors of intraoperative complications of RLS (Table 3). Logistic regression analysis identified several independent risk factors associated with prolonged operative time: neoadjuvant chemotherapy, earlier extrahepatic liver surgery, lesions greater than 50 mm, multiple lesions, bilobar disease, technical major resections, and anatomical major resections (Table 3). Therefore, it was not feasible to develop a reliable DSS based onfactors associated with intraoperative complications, whereas prolonged operative time was deemed the most suitable surrogate marker to assess surgical difficulty and to inform the development of the International RoboLiver DSS. Baseline characteristics of patients undergoing RLS with and without prolonged operative time are presented in the **Supplemental Digital Content 1,** Supplementary Materials, https://links.lww.com/JACS/A687.

**Table 3.** Logistic Regression Analysis of Risk Factors for Intraoperative Complications and Prolonged Operative Time in Robotic Liver Surgery

Variable	Univariable				Multivariable (n = 1,293)		
	OR	95% CI	p Value	β coefficient	Adjusted OR	95% CI	p Value
Intraoperative complication							
BMI >35 kg/m^2^^*^	0.418	0.160–0.898	0.043	-0.798	0.450	0.172–0.978	0.067^†^
Cirrhosis	0.970	0.697–1.334	0.853				
Neoadjuvant chemotherapy	0.877	0.576–1.229	0.526				
Previous extrahepatic surgery	0.747	0.554–1.000	0.052	–0.225	0.798	0.581–1.091	0.161
Previous hepatic surgery	0.670	0.308–1.293	0.268				
Previous open hepatic surgery	0.706	0.111–2.524	0.645				
Malignancy	1.628	1.140–2.374	0.009	0.610	1.841	1.263–2.743	0.002^†^
Size largest lesion, mm							
<30							
30–50	1.080	0.743–1.570	0.685				
>50	1.896	1.345–2.687	<0.001	0.717	2.049^‡^	1.478–2.836^§^	<0.001^‡^^,^^†^
Multiple lesions^‖^	1.118	0.762–1.609	0.558				
Bilobar disease	1.265	0.829–1.882	0.260				
Classification of resection							
Minor							
Technically major	1.367	0.979–1.903	0.065	0.142	1.152	0.807–1.637	0.432
Anatomically major	1.241	0.860–1.777	0.243	-0.140	0.870	0.584–1.282	0.487
Intercept = –2.266							
Prolonged operative time					(n = 1,334)		
BMI >35 kg/m^2^^*^	1.075	0.592–1.861	0.804				
Cirrhosis	0.893	0.675–1.174	0.424				
Neoadjuvant chemotherapy	2.148	1.584 –2.903	<0.001	0.468	1.597	1.117–2.274	0.010^†^
Previous extrahepatic surgery	1.538	1.205–1.964	0.001	0.329	1.389	1.050–1.837	0.021^†^
Previous hepatic surgery	0.890	0.512–1.480	0.666				
Previous open hepatic surgery	0.183	0.010–0.903	0.101				
Malignancy	1.589	1.186–2.152	0.002	0.270	1.310	0.939–1.844	0.116
Size largest lesion, mm							
<30							
30–50	1.110	0.816–1.509	0.507				
>50	1.671	1.246–2.247	0.001	0.376^c^	1.456^‡^	1.083–1.955^‡^	0.013^‡^^,^^†^
Multiple lesions^‖^	2.180	1.616–2.932	<0.001	0.458	1.580	1.117–2.226	0.009^†^
Bilobar disease	2.388	1.702–3.338	<0.001	0.452	1.572	1.056–2.329	0.025^†^
Classification of resection							
Minor							
Technically major	5.871	4.319–8.027	<0.001	0.506	1.659	1.194–2.308	0.003^†^
Anatomically major	1.864	1.358–2.562	<0.001	1.614	5.025	3.622–7.010	<0.001^†^
Intercept = –2.419							

* Missing data for 151 patients.

† Statistically significant.

‡ Reference category ≤50 mm in multivariable analysis.

§ CI.

‖ Missing data for 61 patients.

OR, odds ratio.

A sensitivity analysis demonstrated that neoadjuvant chemotherapy, lesions greater than 50 mm, bilobar disease, technical major resections, and anatomical major resections were still significantly associated with, or showed a trend toward, prolonged operative time after completion of the learning curve (25 procedures), whereas multiple lesions and earlier extrahepatic liver surgery were not (**Supplemental Digital Content 1,** Supplementary Materials, https://links.lww.com/JACS/A687).

Points were assigned to each independent risk factor, resulting in a novel DSS for RLS with a maximum total of 12 points (Table 4). Higher scores correspond to a higher risk of prolonged operative time. The calibrated model exhibited an intercept of 0.00 (95% CI –0.12 to 0.11), consistent with perfect calibration, and a slope of 1.14 (95% CI 0.89 to 1.39), suggesting good model fit, without evidence of significant over- or underfitting. Predicted, observed, and calibrated risk per difficulty score point can be found in the **Supplemental Digital Content 1,** Supplementary Materials (https://links.lww.com/JACS/A687). Scores of 0 to 2 were categorized as low, 3 to 5 as medium, and greater than or equal to 6 as high difficulty (Table 4). The points system had moderate predictive ability for prolonged operative time: AUC 0.719 (95% CI 0.686 to 0.751). Internal validation using 1,000 bootstrap resamples demonstrated an identical bootstrap-corrected AUC: 0.719 (95% bootstrapped CI 0.687 to 0.749), indicating internal stability of the model and confirming moderate discrimination for predicting prolonged operative time.

**Table 4.** Points System for the International RoboLiver Difficulty Scoring System

Factor	Points
Neoadjuvant chemotherapy	
No	0
Yes	1
Previous extrahepatic surgery	
No	0
Yes	1
Largest lesion size, mm	
≤50	0
>50	1
Multiple lesions	
No	0
Yes	1
Bilobar disease	
Unilobar	0
Bilobar	1
Classification of resection	
Minor	0
Technically major	2
Anatomically major	5
Total	…/12

**Figure 2.**
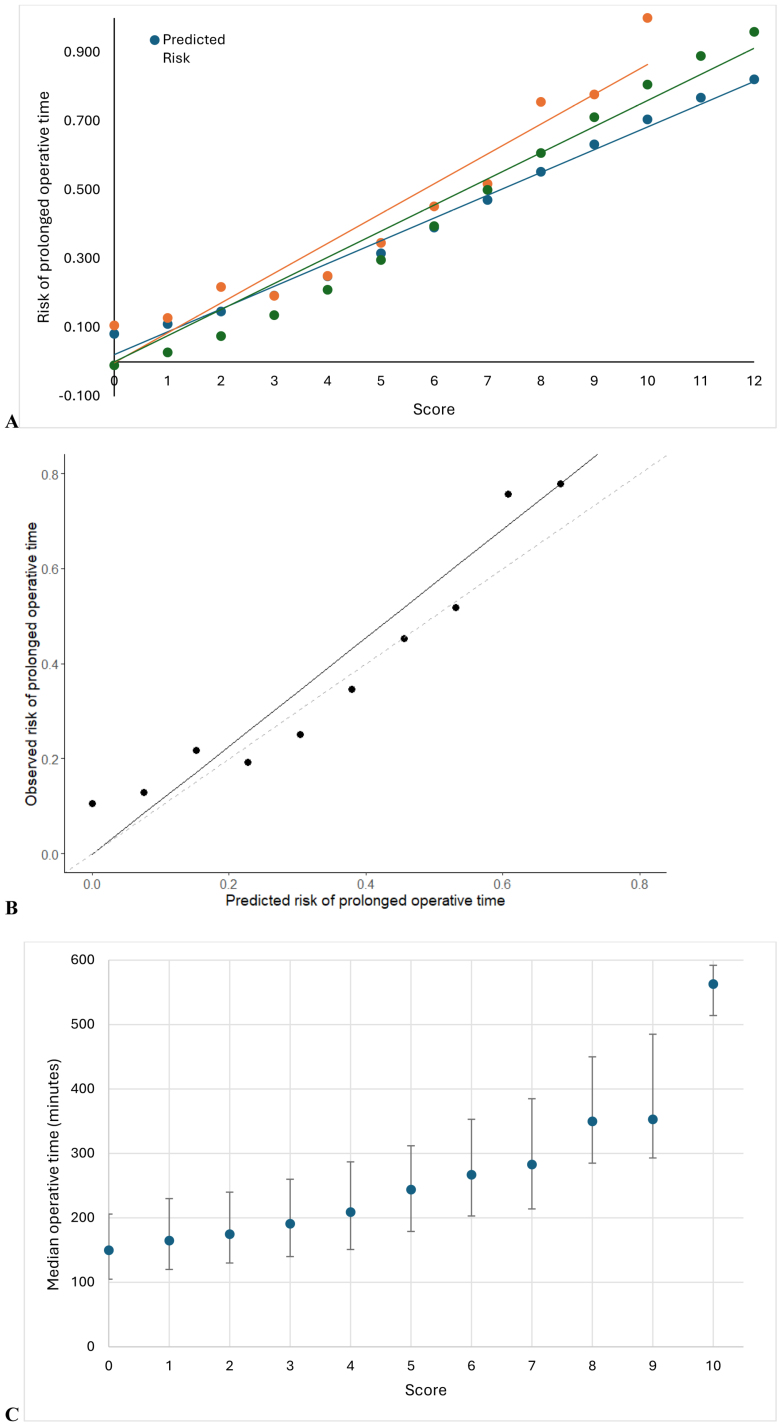
(A) Predicted, observed, and calibrated risk of prolonged operative time using the points system. y = 0.662x + 0.0212 for predicted risk, and y = 0.0867x – 0.002 for observed risk, resulting in a calibrated model with the function y = 0.076x. (B) Calibration plot of the International RoboLiver difficulty scoring system (DSS). (C) Median operative time per score.

Higher International RoboLiver DSS categories were significantly associated with higher intraoperative blood loss, conversion rate, pringle use, pringle duration, intraoperative transfusion use, operative time, length of hospital stay, readmission, and severe morbidity (Table 5). However, the DSS categories were not associated with the rate of intraoperative complications, overall morbidity, mortality, or R0 resection. Figure 2B shows the median operative time for the score.

**Table 5.** Short-Term Perioperative Outcomes per Risk Category of the International RoboLiver Difficulty Scoring System

Variable	Low, N = 726	Moderate, N = 418	High, N = 292	p Value for monotonic increasing trend
Intraoperative blood loss, mL, median (IQR)	100 (50–200)	100 (50–300)	200 (75–400)	<0.001^*^
Intraoperative complications, n (%)	102 (15.0)	64 (16.3)	54 (19.2)	0.114
Conversion, n (%)	12 (1.7)	14 (3.4)	17 (5.9)	<0.001^*^
Pringle application, n (%)	237 (32.7)	197 (47.2)	127 (43.5)	<0.001^*^
Pringle duration, min, median (IQR)	25 (19–37)	30 (20–45)	36 (20–49)	<0.001^*^
Intraoperative transfusion, n (%)	22 (3.1)	29 (7.0)	29 (10.0)	<0.001^*^
Operative time, min, median (IQR)	160 (120–226)	200 (150–279)	295 (220–399)	<0.001^*^
Hospital stay, d, median (IQR)	4 (3–5)	4 (3–6)	5 (4–7)	<0.001^*^
Overall morbidity, n (%)	138 (19.0)	77 (18.4)	64 (21.9)	0.381
Severe morbidity, n (%)	33 (4.6)	23 (5.5)	29 (9.9)	0.002^*^
Readmission, n (%)	37 (5.2)	23 (5.7)	296 (9.0)	0.036^*^
In-hospital/90-d mortality, n (%)	11 (1.5)	4 (1.0)	8 (2.8)	0.298
R0 resection margin for malignant cases only, n (%)	458 (90.5)	284 (87.4)	194 (90.7)	0.774

* Statistically significant.

IQR, interquartile range.

## DISCUSSION

This study confirms that, although complex cases are associated with a higher degree of surgical difficulty, the prospective outcomes and management of these intraoperative challenges can differ from the laparoscopic to robotic approach. A previously developed and validated difficulty score for laparoscopic liver resections, the Southampton DSS, failed to gain validity when applied to the RLS cohort. The predictive ability of the Southampton DSS for intraoperative complications was found to be low (AUC = 0.571), with poor calibration and discrimination.^15^ Although it provides basic stratification, the Southampton DSS is inadequate for precise risk prediction in RLS. Hence, there is a need for a specific RLS DSS that uses prolonged operative time as a measure of difficulty.

Originally developed for LLS, Southampton DSS had a predictive performance with an AUC of 0.677, which was only 0.571 when applied to the present RLS cohort.^6^ This discrepancy likely stems from the fundamental technical differences between RLS and LLS. The Southampton DSS was developed using intraoperative complications, according to the Satava score, as the primary indicator of difficulty.^30^ A large proportion of these intraoperative incidents were conversion to open surgery (41%). It is now obvious that the major benefit offered by robotic systems is a clear reduction in conversion rates.^2,5^ Given the low incidence of conversion in RLS, conversion can no longer be considered a reliable marker of surgical difficulty. This does not imply that when using the robotic approach, resections in the posterior segments or after previous open surgery are as challenging as minor anterior resections or those performed on a virgin abdomen. Surgeons can take advantage of the enhanced dexterity, improved visualization, and greater control offered by the robotic platform, allowing them to take time, manage incidents safely, and complete the procedure with a minimally invasive approach in the most difficult cases.^2,31-33^ These difficult clinical scenarios, including resections in the posterosuperior segments, major anatomical resections, and previous abdominal surgery, were associated with prolonged operative time in RLS.

Although higher risk categories in the International RoboLiver DSS were not associated with intraoperative complications, they were specifically associated with increased blood loss, conversion, Pringle use, hospital stay, and severe postoperative morbidity. This suggests that robotic systems can mitigate the risks of intraoperative complications but cannot completely overcome the fact that these are complex procedures, which are associated with specific risks and complications.

A limited set of predictive factors was significantly associated with intraoperative complications in RLS, limiting the development of a reliable risk stratification tool for this outcome. When prolonged operative time was used as a surrogate marker for procedural difficulty, several predictive factors were identified. Prolonged operative time was used to reflect the overall technical complexity of the procedure. Although operative time may be influenced by various contextual factors, including team experience and case logistics, it remains an objective and reproducible metric that captures multiple aspects of intraoperative difficulty. In RLS, longer operative time typically reflects increased procedural complexity, such as extensive resections and challenging anatomy.

Unsurprisingly, some of the predictive factors overlap with predictors in the Southampton DSS, such as neoadjuvant chemotherapy, lesion size greater than 50 mm, and resection type. However, our model assigns different weights to these variables to reflect their unique relevance in the robotic context. Neoadjuvant chemotherapy may increase procedural difficulty by compromising liver quality through chemotherapy-associated liver injury, thereby making liver dissection, structural recognition, and bleeding management more difficult. However, it is unclear whether the increased difficulty is directly attributable to the effects of neoadjuvant chemotherapy itself or if it is representative of a patient group with more extensive disease, and hence, increased difficulty. Additionally, a lesion size greater than 50 mm was associated with an increase in intraoperative incidence in both RLS and LLS. Larger tumors inherently pose greater technical challenges given their association with nearby structures and the necessity to resect a greater volume of the liver parenchyma. Interestingly, tumors between 30 and 50 mm were associated with increased difficulty in LLS in Southampton DSS but not in RLS. In LLS, mid-sized tumors can pose greater challenges owing to limited instrument maneuverability, particularly in difficult-to-access segments, whereas instrument dexterity in RLS mitigates these challenges. It is unsurprising that resections involving the posterosuperior segments and major resections significantly increase procedural difficulty, given the need for greater liver mobilization, challenging anatomical positioning, and limited surgical access.

A history of previous extrahepatic surgery, bilobar disease, and multiple lesions was predictive of prolonged operative time but was not associated with intraoperative complications in RLS. These factors are also not associated with intraoperative complications in laparoscopy according to the Southampton DSS. It is difficult to determine whether these factors were associated with prolonged operation in laparoscopic resections in the Southampton DSS, as this was not specifically analyzed. However, their association with prolonged operative time in robotic resection reflects the expected higher difficulty. In fact, dissecting adhesions and repositioning trocars to reach all lesions or a different lobe are time-consuming tasks; however, the safety of these highly demanding robotic procedures has been demonstrated, as no association was found between these procedures and complications. This finding suggests that surgeons trained in RLS can manage these technical challenges without compromising patient safety, thereby supporting the application of broad selection criteria when determining candidates for RLS.

Interestingly, although a history of open liver resection has been associated with increased intraoperative complications in LLS, this was not observed in RLS. This may reflect the enhanced ability of RLS to more safely manage intra-abdominal adhesions from previous surgeries.^31^ However, previous abdominal surgery was still associated with a prolonged operative time in RLS. Likewise, neoadjuvant chemotherapy and lesion size between 30 and 50 mm did not appear to increase the risk of intraoperative complications in RLS, whereas according to Southampton DSS, they did in LLS. The technical advantages of RLS help tackle these specific challenges safely, which may support less stringent patient selection during the early phases of the RLS learning curve.

The Southampton DSS tended to overestimate the risk of intraoperative complications in RLS in patients with higher difficulty scores. This further supports the notion that RLS enhances the feasibility and safety of complex liver resections. However, it is important to acknowledge that the lower incidence of intraoperative complications in these patients may partly reflect the later adoption of RLS, which allows surgeons to build on the accumulated experience gained through LLS. Many surgeons transitioning to robotic platforms have undergone structured training programs that have progressed gradually from simple to complex resections.^34^ This stepwise learning process, coupled with increased awareness of factors associated with MILS complexity in the robotic era, likely contributed to the lower incidence of intraoperative complications.^30^ For this reason, we based our novel difficulty score on prolonged operative time, as it was hypothesized to be a more sensitive indicator of technical difficulty. Knowing the expected duration of the procedures supports better operating room planning.

Our findings align with those of recent studies^12,13,35,36^ that highlight the limited applicability of laparoscopic-based difficulty scores for RLS. Although these studies were often based on small, single-center cohorts, they assessed the applicability of various laparoscopic-based DSSs (Southampton,^6^ IWATE,^7^ and Institut Mutualiste Mountsouris^8^) for RLS. They found that these DSSs performed suboptimally when applied to the RLS, underscoring the need for robot-specific DSSs.

Recently, Sucandy and colleagues^18^ proposed the Tampa Difficulty Score, which includes factors associated with operative time or blood loss during RLS. Neoadjuvant chemotherapy, tumor location (Couinaud segment), tumor size (less than 3 cm or greater than or equal to 3 cm), type (benign or malignant), extent of resection (according to the Brisbane classification system^23^), portal lymphadenectomy, and biliary reconstruction were identified as factors significantly associated with procedural difficulty. Several of these factors overlapped with those identified in the current study. However, it was developed in a single-center setting with a small patient sample, which limits its generalizability. In contrast, our multicenter dataset and larger cohort enhanced the external validity of our findings. Additionally, by including procedures performed during the learning curve, we sought to enhance the generalizability of this DSS to less-experienced robotic surgeons. Importantly, we aimed to create a clinically applicable risk stratification tool for predicting a prolonged operative time. To promote usability, we simplified certain factors; for example, tumor location was grouped into anterolateral vs posterosuperior segments, and resection extent was categorized as minor vs major. Additionally, our approach intentionally excluded highly specific procedural elements such as lymphadenectomy and biliary reconstruction. By excluding cases of major concurrent surgeries, we provided a score tailored specifically to the technical complexity of liver resection. Clinically, International RoboLiver DSS can be used to support preoperative patient selection and planning by aligning surgeon or team assignment and perioperative logistics with anticipated technical difficulty. In practice, higher International RoboLiver DSS categories may prompt consideration of stepwise case selection, particularly during the learning curve, allocation to more experienced teams or proctoring, and intensified preparation for complex scenarios. In addition, the International RoboLiver DSS provides a standardized framework for patient counseling about anticipated procedural complexity. The International RoboLiver DSS is intended to complement, not replace, clinical judgment.

Nevertheless, this study has several limitations. First, the retrospective design and the exclusion of patients with incomplete data required to calculate the Southampton DSS score introduced selection bias. Second, the complexity of liver resection in our novel scoring system is based on a surrogate outcome, namely, prolonged operative time, which may not always directly correspond to procedural difficulty. Developing difficulty scores based on the surgeon’s perceived effort with prospective data collection could help more accurately identify the factors contributing to procedural complexity, instead of using a surrogate outcome. Third, time bias is inherent because of the later adoption of RLS, resulting in greater awareness of the risks and factors contributing to MILS difficulty during its implementation, as discussed in detail previously. Fourth, although this study included procedures from the early and late phases of the RLS learning curve, several surgeons had earlier experience with LLS, which may have facilitated the transition to RLS. Therefore, although the findings may reflect a range of robotic expertise, they may not be fully generalizable to centers with limited experience in MILS. Selection bias is possible because case assignment to the robotic approach was based on surgeon discretion, which may be influenced by experience. Finally, these findings have not yet undergone external validation. Nevertheless, the transparent methodology and comprehensive reporting provided in the current study are intended to enable and encourage independent validation in future research.

DSSs are a vital tool in clinical practice and research. Clinically, they support preoperative planning, guide case selection, and help anticipate the technical challenges. They also aid in matching patients to surgeons based on their experience, such as when selecting training cases for novice robotic surgeons. Additionally, recognizing procedures likely to involve prolonged operative times enables more efficient operative scheduling. Standardized DSSs help stratify procedures based on difficulty, enabling consistent reporting and facilitating comparisons across studies. Although the International RoboLiver DSS only demonstrates moderate discrimination (AUC 0.719), this level of predictive performance remains clinically meaningful in the preoperative context. Even moderate risk separation can support pragmatic decision-making, facilitating appropriate case selection between open, robotic, and laparoscopic approaches, optimizing surgical planning, and supporting patient counseling about anticipated complexity. Ultimately, the DSS serves as a support tool for surgical decision-making; however, it should not substitute for clinical judgment.

Artificial intelligence could help refine future DSSs for RLS through in-depth analysis of both preoperative imaging and intraoperative videos. Artificial intelligence has shown promise in identifying predictive patterns, allowing large-scale analysis of preoperative 3-dimensional liver modeling and surgical video data.^37-39^ The integration of these tools could yield more robust surgical DSSs.

In conclusion, although Southampton DSS provides a useful foundation for case stratification in LLS, it lacks precision when applied to RLS. The International RoboLiver DSS represents a significant step forward for the accurate prediction of surgical complexity in RLS. Nevertheless, external validation across diverse clinical settings and varying levels of surgical expertise is essential to confirm its broader use.

## Author Contributions

Data curation: El Adel, Pilz da Cunha

Formal analysis: El Adel, Pilz da Cunha, van Dieren

Methodology: El Adel, Pilz da Cunha, van Dieren

Project administration: El Adel

Writing – original draft: El Adel, Pilz da Cunha, Swijnenburg, Abu Hilal

Conceptualization: Pilz da Cunha, Swijnenburg, Abu Hilal

Validation: Pilz da Cunha

Writing – review & editing: Pilz da Cunha, Liu, Sucandy, Di Benedetto, D’Hondt, Vrochides, Croner, Sparrelid, Ratti, Geller, Gallagher, Goh, Jovine, Ahmad, Ruzzenente, Campos, White, Efanov, Memeo, Cillo, Alzoubi, Schmelzle, Alseidi, Swijnenburg, Abu Hilal

Investigation: van Dieren

Supervision: Swijnenburg, Abu Hilal

## Supplementary Material


