# Supplementary material for: Validation of the Southampton Difficulty Scoring System in Robotic Liver Surgery and Development of the International RoboLiver Difficulty Scoring System
Source: J Am Coll Surg. 2026 May 1;243(3):626–39. doi: 10.1097/XCS.0000000000001978 (PMC13460327; doi:10.1097/XCS.0000000000001978)
Supplement: Supplementary file 1 [file xcs-243-626-s001.pdf]

Supplementary Materials

Supplementary Table 1: Baseline characteristics per risk category of the Southampton DSS

	**Overall**	**Low**	**Moderate**	**High**	**Extremely High**	**p-value**
	**n=1497**	**n=317**	**n=613**	**n=546**	**n=21**	
**Age at operation, median [IQR]**	62 [52 - 70]	60 [52 - 69]	62 [51 - 70]	63 [54 - 71]	61 [50 - 68]	0.203
**Male sex, n (%)**	854 (57.1)	159 (50.2)	331 (54.1)	350 (64.1)	14 (66.7)	**<0.001**
**BMI, median [IQR]**	25.4 [23.0 - 29.1]	26.0 [23.5 - 30.0]	25.8 [23.0 - 29.5]	25.0 [22.9 - 28.0]	25.3 [23.2 - 27.9]	**0.024**
**ASA Grade ≥3, n (%)**	544 (36.9)	125 (39.9)	216 (35.8)	192 (35.7)	11 (52.4)	0.257
**Cirrhosis, n (%)**	394 (26.3)	93 (29.3)	152 (24.8)	149 (27.3)	0 (0.0)	**0.018**
**Neoadjuvant chemotherapy, n (%)**	245 (16.4)	2 (0.6)	129 (21.0)	93 (17.0)	21 (100.0)	**<0.001**
**Previous extrahepatic abdominal surgery, n (%)**	641 (42.8)	126 (39.7)	283 (46.2)	218 (39.9)	14 (66.7)	**0.013**
**Previous hepatic surgery, n (%)**	84 (5.6)	21 (6.6)	21 (3.4)	38 (7.0)	4 (19.0)	**0.002**
Open	17 (1.1)	0 (0.0)	1 (0.2)	15 (2.7)	1 (4.8)	0.206
**Histological diagnosis, n (%)**						**<0.001**
CRLM	330 (22.0)	29 (9.1)	151 (24.6)	136 (24.9)	14 (66.7)	
HCC	542 (36.2)	82 (25.9)	202 (33.0)	254 (46.5)	4 (19.0)	
CCA	105 (7.0)	18 (5.7)	36 (5.9)	49 (9.0)	2 (9.5)	
Gallbladder carcinoma	42 (2.8)	20 (6.3)	21 (3.4)	1 (0.2)	0 (0.0)	
Non-CRLM	31 (2.1)	7 (2.2)	35 (5.7)	31 (5.7)	1 (4.8)	
Other malignancy	74 (4.9)	7 (2.2)	17 (2.8)	7 (1.3)	0 (0.0)	
Benign	373 (24.9)	154 (48.6)	151 (24.6)	68 (12.5)	0 (0.0)	
**Size largest lesion in mm, median [IQR]**	35 [22 - 60]	20 [15 - 25]	36 [23 - 60]	46 [33 - 70]	70 [60 - 85]	**<0.001**
**Multiple lesions, n (%)**	250 (17.4)	29 (10.6)	98 (16.3)	115 (21.3)	8 (38.1)	**<0.001**
**Bilobar disease, n (%)**	183 (12.2)	39 (12.3)	58 (9.5)	77 (14.1)	9 (42.9)	**<0.001**
**Classification of resection, n (%)**						**<0.001**
Minor	714 (47.7)	294 (92.7)	395 (64.4)	24 (4.4)	1 (4.8)	
Technically major	445 (29.7)	23 (7.3)	204 (33.3)	218 (39.9)	0 (0.0)	
Anatomically major	338 (22.6)	0 (0.0)	14 (2.3)	304 (55.7)	20 (95.2)	

Supplementary Table 2: Predicted observed and calibrated risk of prolonged operative time using the International RoboLiver DSS

Score	n	Predicted Risk	Observed Risk	Calibrated Risk[a]	Calibrated predicted risk[b]	Category
0	227	0.082	0.106	-0.010	0	Low
1	265	0.110	0.128	0.027	0.076	
2	234	0.147	0.218	0.075	0.152	
3	234	0.193	0.192	0.136	0.228	
4	99	0.249	0.250	0.210	0.304	Medium
5	85	0.316	0.346	0.297	0.380	
6	144	0.391	0.452	0.395	0.456	
7	85	0.471	0.518	0.500	0.532	
8	42	0.553	0.756	0.608	0.608	High
9	18	0.632	0.778	0.711	0.684	
10	3	0.705	1.000	0.806	0.760	
11	0	0.769		0.890	0.836	
12	0	0.822		0.960	0.912	

[a] calculated from using 0.0867 * ((predicted risk - 0.0212) / 0.0662) - 0.089, [b]calculated from graphical function y=0.076x of calibrated risk

Supplementary Table 3: Baseline characteristics of patients undergoing RLS with and without prolonged operative time.

	**No prolonged operative time**	**Prolonged operative time**	**p-value**
	**n=1037**	**n=349**	
**Age at operation, median [IQR]**	61 [51 - 70]	62 [53 - 71]	0.302
**Male sex, n (%)**	576 (55.6)	214 (61.3)	0.062
**BMI, median [IQR]**	25.1 [22.9 - 28.7]	26.1 [23.5 - 29.4]	**0.003**
**ASA Grade ≥3, n (%)**	330 (32.4)	159 (46.0)	**<0.001**
**Cirrhosis, n (%)**	287 (27.7)	89 (25.5)	0.424
**Neoadjuvant chemotherapy, n (%)**	137 (13.2)	86 (24.6)	**<0.001**
**Previous extrahepatic abdominal surgery, n (%)**	410 (39.5)	175 (50.1)	**0.001**
**Previous hepatic surgery, n (%)**	63 (6.1)	19 (5.4)	0.666
Open	16 (1.5)	1 (0.3)	0.065
**Histological diagnosis, n (%)**			**0.010**
CRLM	209 (20.2)	89 (25.5)	
HCC	384 (37.0)	131 (37.5)	
CCA	59 (5.7)	33 (9.5)	
Gallbladder carcinoma	27 (2.6)	8 (2.3)	
Non-CRLM	15 (1.4)	3 (0.9)	
Other malignancy	55 (5.3)	17 (4.9)	
Benign	288 (27.8)	68 (19.5)	
**Size largest lesion in mm, median [IQR]**	35 [22 - 60]	42 [25 - 65]	**0.002**
**Multiple lesions, n (%)**	142 (14.3)	92 (26.7)	**<0.001**
**Bilobar disease, n (%)**	97 (9.4)	69 (19.8)	**<0.001**
**Classification of resection, n (%)**			**<0.001**
Minor	557 (53.7)	92 (26.4)	
Technically major	315 (30.4)	97 (27.8)	
Anatomically major	165 (15.9)	160 (45.8)	

Supplementary Table 4: Baseline characteristics per risk category of the International RoboLiver DSS

	**Low**	**Moderate**	**High**	**p-value**
	**n=726**	**n=418**	**n=292**	
**Age at operation, median [IQR]**	61 [52 - 70]	63 [54 - 71]	63 [51 - 71]	0.118
**Male sex, n (%)**	412 (56.8)	247 (59.1)	167 (57.2)	0.750
**BMI, median [IQR]**	25.5 [23.1 - 29.2]	25.5 [23.0 - 29.0]	25.2 [22.9 - 28.5]	0.558
**ASA Grade ≥3, n (%)**	256 (35.7)	152 (37.1)	118 (40.8)	0.314
**Cirrhosis, n (%)**	239 (32.9)	88 (21.1)	60 (20.6)	**<0.001**
**Neoadjuvant chemotherapy, n (%)**	65 (9.0)	100 (23.9)	77 (26.4)	**<0.001**
**Previous extrahepatic abdominal surgery, n (%)**	223 (30.7)	232 (55.5)	156 (53.4)	**<0.001**
**Previous hepatic surgery, n (%)**	39 (5.4)	21 (5.0)	21 (7.2)	0.424
Open	13 (1.8)	3 (0.7)	1 (0.3)	0.089
**Histological diagnosis, n (%)**				**<0.001**
CRLM	104 (14.3)	140 (33.5)	85 (29.1)	
HCC	303 (41.7)	138 (33.0)	97 (33.2)	
CCA	54 (7.4)	21 (5.0)	28 (9.6)	
Gallbladder carcinoma	20 (2.8)	5 (1.2)	0 (0.0)	
Non-CRLM	19 (2.6)	7 (1.7)	5 (1.7)	
Other malignancy	30 (4.1)	33 (7.9)	11 (3.8)	
Benign	196 (27.0)	74 (17.7)	66 (22.6)	
**Size largest lesion in mm, median [IQR]**	32 [20 - 50]	35 [23 - 55]	60 [30 - 85]	**<0.001**
**Multiple lesions, n (%)**	1 [1 - 1]	1 [1 - 2]	1 [1 - 2]	**<0.001**
**Bilobar disease, n (%)**	52 (7.2)	117 (28.0)	81 (27.7)	**<0.001**
**Classification of resection, n (%)**				**<0.001**
Minor	616 (84.8)	52 (12.4)	0 (0.0)	
Technically major	110 (15.2)	316 (75.6)	12 (4.1)	
Anatomically major	0 (0.0)	50 (12.0)	280 (95.9)	

Supplementary Table 5: Sensitivity analysis of factors associated with operative time after the learning curve

Prolonged operative time							
	Univariable			Multivariable			
**Variable**	**OR**	**95% CI**	**p-value**	**B**	**aOR**	**95% CI**	**p-value**
BMI >35	1.700	0.902–3.074	0.088	0.608	1.837	0.879–3.711	0.096
Cirrhosis	0.784	0.553–1.099	0.165	–	–	–	–
Neoadjuvant chemotherapy	2.470	1.689–3.587	<0.001	0.704	2.022	1.267–3.207	0.003[b]
Previous extrahepatic surgery	1.621	1.193–2.203	0.002	0.254	1.290	0.888–1.870	0.180
Previous hepatic surgery	1.022	0.535–1.836	0.943	–	–	–	–
Previous open hepatic surgery	0.262	0.014–1.315	0.197	–	–	–	–
Malignancy	1.424	0.982–2.105	0.069	0.276	1.318	0.847–2.092	0.229
Size largest lesion, mm							
<30mm							
30-50mm	0.885	0.594–1.311	0.543	–	–	–	–
>50mm	1.656	1.157–2.377	0.006	0.360	1.433	0.967–2.119	0.07 2[c]
Multiple lesions	1.911	1.305–2.771	<0.001	0.165	1.180	0.732–1.876	0.491
Bilobar disease	2.393	1.602–3.541	<0.001	0.502	1.653	1.008–2.686	0.044 [b]
Classification of resection							
Minor							
Technically Major	2.284	1.482–3.554	<0.001	0.753	2.122	1.319–3.452	0.002[b]
Anatomically Major	7.569	5.061–11.526	<0.001	1.893	6.640	4.229–10.640	<0.001 [b]

[a]reference category ≤50mm in multivariable analysis.[b]statistically significant [c]trend towards significance. aOR, adjusted odds ratio; B, coefficient; CI, confidence interval; OR, odds ratio
